# Improved Productivity of *Streptomyces mobaraensis* Transglutaminase by Regulating Zymogen Activation

**DOI:** 10.3389/fbioe.2022.878795

**Published:** 2022-04-14

**Authors:** Xiaoqiang Yin, Shengqi Rao, Jingwen Zhou, Guocheng Du, Jian Chen, Song Liu

**Affiliations:** ^1^ National Engineering Research Center for Cereal Fermentation and Food Biomanufacturing, Jiangnan University, Wuxi, China; ^2^ Science Center for Future Foods, Jiangnan University, Wuxi, China; ^3^ School of Biotechnology, Jiangnan University, Wuxi, China; ^4^ College of Food Science and Engineering, Yangzhou University, Yangzhou, China; ^5^ Jiangsu Provincial Engineering Research Center for Bioactive Product Processing, Jiangnan University, Wuxi, China

**Keywords:** transglutaminase, *Streptomyces mobaraensis*, NH_4_^+^, zymogen activation, productivity

## Abstract

*Streptomyces mobaraensis* transglutaminase (TGase) is extracellularly expressed as a zymogen and then activated by TGase-activating protease (TAP). In this study, we reported the strategy for improving TGase production *via t*he regulation of TAP activity in *S. mobaraensis*. First, we analyzed the effects of three inorganic nitrogen sources on TGase production. With 30 mM nitrogen content, the time to the peak of TGase activity induced by (NH_4_)_2_SO_4_ or NH_4_Cl was 72 h, 12 h earlier than that of the fermentation without adding NH_4_^+^. SDS-PAGE analysis indicated that NH_4_^+^ accelerated the TGase activation in *S. mobaraensis*. Then, we examined the effect of NH_4_^+^ on TAP biosynthesis using a TGase-deficient *S. mobaraensis* strain. It showed that NH_4_^+^ enhanced the TAP activity at the early stage of the fermentation, which was dependent on the concentration and time of NH_4_^+^ addition. Last, the yield and productivity of *S. mobaraensis* TGase were increased by 1.18-fold and 2.1-fold, respectively, when optimal NH_4_^+^ addition (60 mM and 12 h) was used. The fermentation period was shortened from 84 to 48 h. The NH_4_^+^ addition also increased the storage stability of crude enzyme at room temperature. These findings will benefit the TGase production and its activation mechanism in *S. mobaraensis*.

## Introduction

Transglutaminase (TGase, EC 2.3.2.13) belongs to the transferases family that introduces covalent cross-links in proteins between glutamine residues and primary amines through an acyl-transfer reaction (Akbari et al., 2021). Due to the unique catalytic reaction, TGase has been exploited to improve the texture properties of protein-based foods (Miwa, 2020). Recently, it has also exhibited application potential in pharmacological production, textile industry, and leather processing (Zhu and Tramper, 2008; Duarte et al., 2019; Doti et al., 2020). In contrast to the enzymes extracted from animals and plants, *Streptomyces mobaraensis* TGase is a Ca^2+^-independent enzyme and easier to be produced on a large scale (Ando et al., 1989). Although *Streptomyces* TGases have been expressed in a variety of heterologous hosts, only *S. mobaraensis* TGase is approved as generally recognized as safe (GRAS) (Yin et al., 2021). These advantages benefit the TGase application and make *S. mobaraensis* fermentation become the main source of commercial TGase products (Santhi et al., 2017). Therefore, it is desirable to improve the TGase production by *S. mobaraensis*.

Since the discovery of *Streptomyces* TGase in 1989, isolating novel TGase-producing strains (Ceresino et al., 2018) and screening high-yielding mutant strains have long been used for enhancing TGase production (Jiang et al., 2017; Yin et al., 2021). On the other hand, the media composition and fermentation process were critical for TGase biosynthesis (Akbari et al., 2021). Transcription analysis indicated that protease, CTAB, and MgCl_2_ were proven to promote TGase expression in *S. mobaraensis* (Fatima and Khare, 2021). To improve the economic efficiency, TGase fermentation was conducted using agricultural wastes, such as wheat bran and non-commercial potatoes (Guerra-Rodriguez and Vazquez, 2014; Fatima et al., 2019). Compared to genetic modification, these “non-genetic” strategies are much easier to be accepted for food industries. To date, the highest TGase activity (19.7 U/mL) in *S. mobaraensis* was achieved by random mutagenesis based on atmospheric and room-temperature plasma mutagenesis and flow cytometry technology (Yin et al., 2021). However, the fermentation period of *Streptomyces* TGase usually reached 72–96 h or even over 10 days (Akbari et al., 2021). Thus, reducing the fermentation period is crucial for enhancing TGase productivity in *S. mobaraensis*.

In *S. mobaraensis*, TGase is exported in the form of inactive zymogen (pro-TGase) and then fully activated into a mature form by its endogenous metalloprotease (TAMP) and AP-specific tri/tetrapeptidyl aminopeptidase within the next 2 days (Zotzel et al., 2003a; Zotzel et al., 2003b). This activation process is regulated by a *Streptomyces subtilisin* inhibitor (SSTI), which could inhibit TAMP activity (Juettner et al., 2020). Therefore, improving the activation process is an important strategy to reduce the fermentation period. It has been demonstrated that *in vitro* protease addition reduced the fermentation period of the *Streptomyces hygroscopicus* TGase by 18% (Zhang et al., 2010). By inducing the overexpression of total protease, metalloprotease, and serine protease, MgCl_2_ can also accelerate the activation of pro-TGase (Zhang et al., 2012). However, protease addition is not cost-effective, while excessive MgCl_2_ is detrimental to cell growth (Zhang et al., 2012). This activation was effective in the context of a small amount of zymogen. In addition, cetyltrimethylammonium bromide is predicted to inactivate the protease inhibitor, resulting in improved activation (Zhang et al., 2008). To be noted, all the protease-mediated activations were investigated using the *Streptomyces* strains with relatively low TGase production.

In this study, we first investigated the effects of three inorganic nitrogen sources on the fermentation period of smY 2019 (a robust variant of *S. mobaraensis* DSM40587) (Yin et al., 2021), determining that NH_4_^+^ could improve TGase activation. Based on a reliable measure for TAP activity using a TGase-deficient *S. mobaraensis* strain smY2019∆*tg*, the changes of TAP activity during fermentation were precisely characterized. It was shown that NH_4_^+^ enhanced TAP activity, dependent on the concentration and time of NH_4_^+^ added. Finally, TGase productivity was significantly improved by regulating zymogen activation in smY 2019.

## Materials and Methods

### Strains and Plasmids

*S. mobaraensis* smY2109 and smY2019∆*tg* were used to study TGase and TAP, respectively (Yin et al., 2021). The plasmid pET-22b (+) and *Escherichia coli* BL21 (DE3) were used for expressing pro-TGase.

### Culture Conditions for *S. mobaraensis*

The spore culture on the GYM agar medium and seed culture in shake flasks were performed as described in the previous study (Yin et al., 2021). The composition of the basal fermentation medium was as follows: 2% glycerol, 2% peptone, 0.5% yeast extract, 2% soya flour, 0.4% K_2_HPO_4_, 0.2% KH_2_PO_4_, and 0.2% MgSO_4_. To study the influence of different inorganic nitrogen sources on the TGase fermentation period, NaNO_3_, (NH_4_)_2_SO_4_, and NH_4_Cl were added to the basal fermentation medium in the same total nitrogen content (30 mM final concentration).

### Construction, Expression, and Purification of the Pro-TGase in *E. coli*

The gene fragment of pro-TGase was amplified from the *S. mobaraensis* smY2019 genome by PrimeSTAR GXL DNA Polymerase (TaKaRa, Dalian, China) using the primer pair ptg-F (CATGCCATGGGCAGCGGCACCGGGGAAGAGAAGAG)/ptg-R (CCG​CTC​GAG​CGG​CCA​GCC​CTG​TGT​CAC​CTT​GTC​G) and cloned into the Nco I-Xho I sites of pET-22b (+), generating the pro-TGase expression plasmid pET-22b/ptg. The plasmid pET-22b/ptg was introduced into *E. coli* BL21 (DE3). The recombinant *E. coli* strain was inoculated into a Luria–Bertani medium containing 100 μg/ml ampicillin for seed culture at 37°C for 12 h. Then, 1-ml seed cultures were transferred into a 50-ml terrific broth (TB) medium containing the same amount of antibiotics and further cultivated at 37°C. At an OD_600_ of 0.8, the cells were induced by adding the inducer isopropyl beta-D-1-thiogalactopyranoside (400 μM, final concentration). Growth was continued at 20°C for up to 40 h. The culture supernatant was subjected to affinity purification using the His-Trap column (GE Healthcare, NY, United States). The pro-TGase was eluted with elution buffer (50 mM Tris-HCl, 50 mM NaCl, and 150 mM imidazole, pH 8.0) and dialyzed against dialysis buffer (50 mM Tris-HCl, pH 8.0). Protein concentration of purified pro-TGase was determined by using the BCA protein assay kit (Beyotime, Shanghai, China). The samples were diluted to 0.5 mg/ml of protein concentrations and used as the substrate for TAP activity measurement.

### TGase-Activating Protease Activity Analysis

For detecting the protease activity, the activation reaction was initiated by mixing the purified pro-TGase (0.5 mg/ml) with an equal volume of the culture supernatant of smY2019∆*tg*. One unit of the TAP toward the pro-TGase was defined as the amount of enzyme needed to generate one unit of mature TGase per hour at 30°C.

### Dry Cell Mass Determination

The biomass of *S. mobaraensis* was measured by using the dry cell weight (DCW) method. *S. mobaraensis* cells were harvested by centrifugation (5,000 × *g*, 15 min) from 10 ml fermentation broth. After washing with sterile water three times, the cell pellets were dried at 105°C until they had a constant weight.

### TGase Activity Analysis

According to the previous report (Yin et al., 2021), the colorimetric method was conducted to measure TGase activity using N-CBZ-Gln-Gly (Sigma-Aldrich, Shanghai, China) as the substrate. One unit of TGase activity is defined as the amount of enzyme needed to generate 1 μmol of hydroxamate per min at 37°C.

### SDS-PAGE Analysis

SDS-PAGE analysis was performed to separate proteins on a 10% running gel, which was visualized after staining with Coomassie Brilliant Blue R250.

### Statistical Analysis

The logistic function was used to fit the curve of the specific production rate by OriginLab 2018 software (OriginLab Corporation, Northampton, United States). First-order kinetics was applied to calculate the specific production rate as follows:

$$\text{specific production rate}=\;\frac{1}{X}\text{×}\frac{\text{dp}}{\text{dt}}\text{ ×}1000\text{,}$$ (1)

where X is DCW (g/L); t is culture time (h); and P is TGase activity (U/mL).

All experiments were carried out in triplicate at least.

## Results and Discussion

### Effects of Inorganic Nitrogen Sources on TGase Production

**FIGURE 1:**
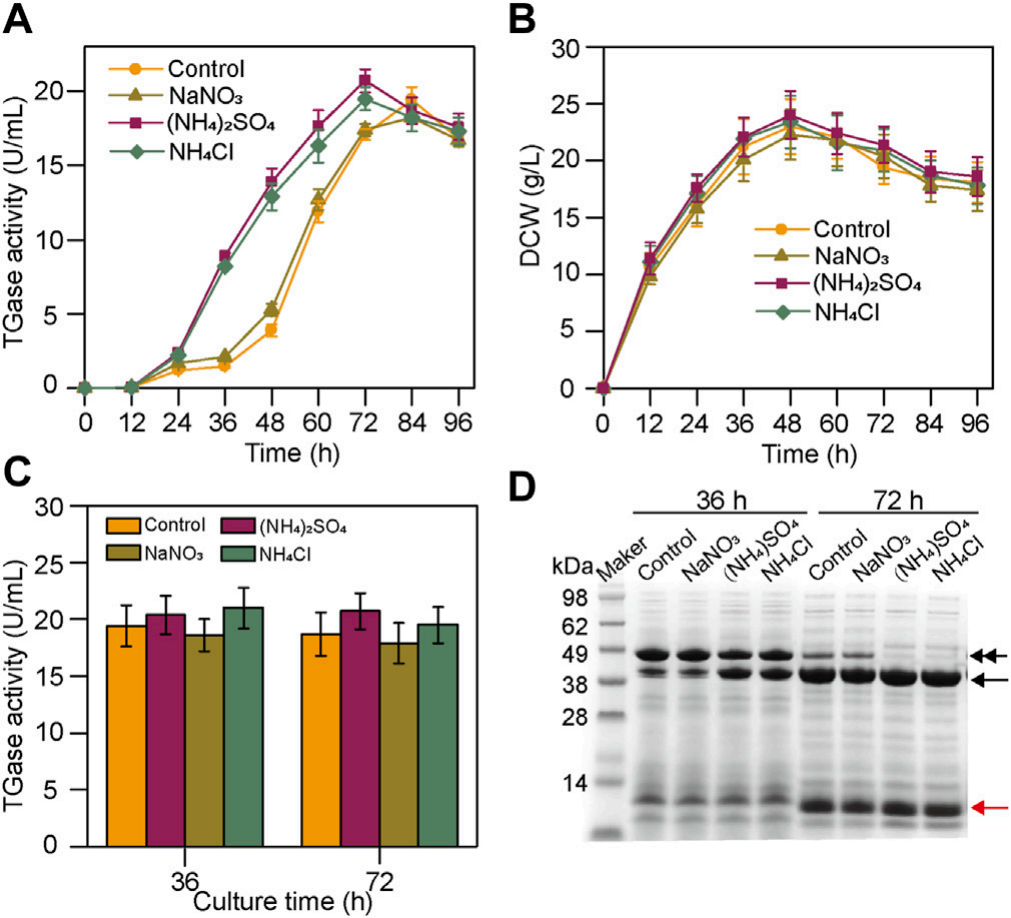
Effects of an inorganic nitrogen source on TGase production by smY 2019. **(A)** Time course of TGase production with the addition of different inorganic nitrogen sources. **(B)** Time course of biomass. **(C)** TGase activity assay after the activation by dispase. **(D)** SDS-PAGE analysis of the culture supernatants. The pro-TGase and TGase bands are indicated with black double and single arrows, respectively. The SSTI bands were identified using MALDI-TOF/MS (data not shown) and are indicated with red arrows. Each fermentation process was performed in a 250-ml flask containing 30 ml of the fermentation medium added with 30 mM of inorganic nitrogen at 30°C and 220 rpm. In the control experiment, no inorganic nitrogen source was added.

Nitrogen source is critical for cell growth and product biosynthesis. The ectoine production was improved by optimizing the type and quantity of the nitrogen sources (Zhang et al., 2022). However, previously, there were very few reports on the biosynthesis of TGase using inorganic nitrogen sources. In the present study, based on the basal fermentation medium, we analyzed the effects of three inorganic nitrogen sources at a constant concentration (30 mM) on the production of TGase by the *S. mobaraensis* smY 2019. As shown in Figure 1A, the cultivation without the inorganic nitrogen sources rapidly accumulated the TGase after 36 h, and the maximal enzyme activity was at 84 h. In the case of (NH_4_)_2_SO_4_ and NH_4_Cl, the rapid TGase production started at 24 h, and the peak value of the enzyme activity occurred at 72 h, 12 h earlier than the control. In contrast, NaNO_3_ did not affect the TGase biosynthesis (Figure 1A). The cell growth of smY2019 was not significantly affected by adding the inorganic nitrogen sources (Figure 1B), suggesting that the reduced TGase production period is not due to cell growth changes. As we all know, TGase was secreted as inactive pro-TGase and then transformed into active mature TGase (Zotzel et al., 2003a). To understand the reason for the accelerated TGase biosynthesis, the culture supernatant of each condition was taken at 36 h and 72 h and subjected to SDS-PAGE analysis. For the samples taken at 36 h, both pro-TGase (43 kDa) and TGase (38 kDa) bands could be seen in all cases (Figure 1D). The sample with NaNO_3_ added had similar protein bands with the control, with thick bands of pro-TGase and thin bands of TGase, while the addition of (NH_4_)_2_SO_4_ or NH_4_Cl had more thick bands of TGase. When fermented in the medium with (NH_4_)_2_SO_4_ or NH_4_Cl for 72 h, the pro-TGase bands were completely converted into TGase bands. However, in addition to the TGase bands, thin pro-TGase bands were still detected at 72 h in the case of control and NaNO_3_ (Figure 1D). After the *in vitro* activation with dispase, all the samples at 36 h and 72 h shared similar TGase activities (18.6–21.1 U/mL) (Figure 1C). These findings indicated that the addition of (NH_4_)_2_SO_4_ and NH_4_Cl significantly influences *S. mobaraensis* TGase activation instead of pro-TGase expression. However, Na_2_SO_4_ and NaCl did not improve the TGase activation (data not shown), suggesting the critical role of NH_4_^+^.

### Effects of NH_4_^+^ on the Activity of TAP

**FIGURE 2:**
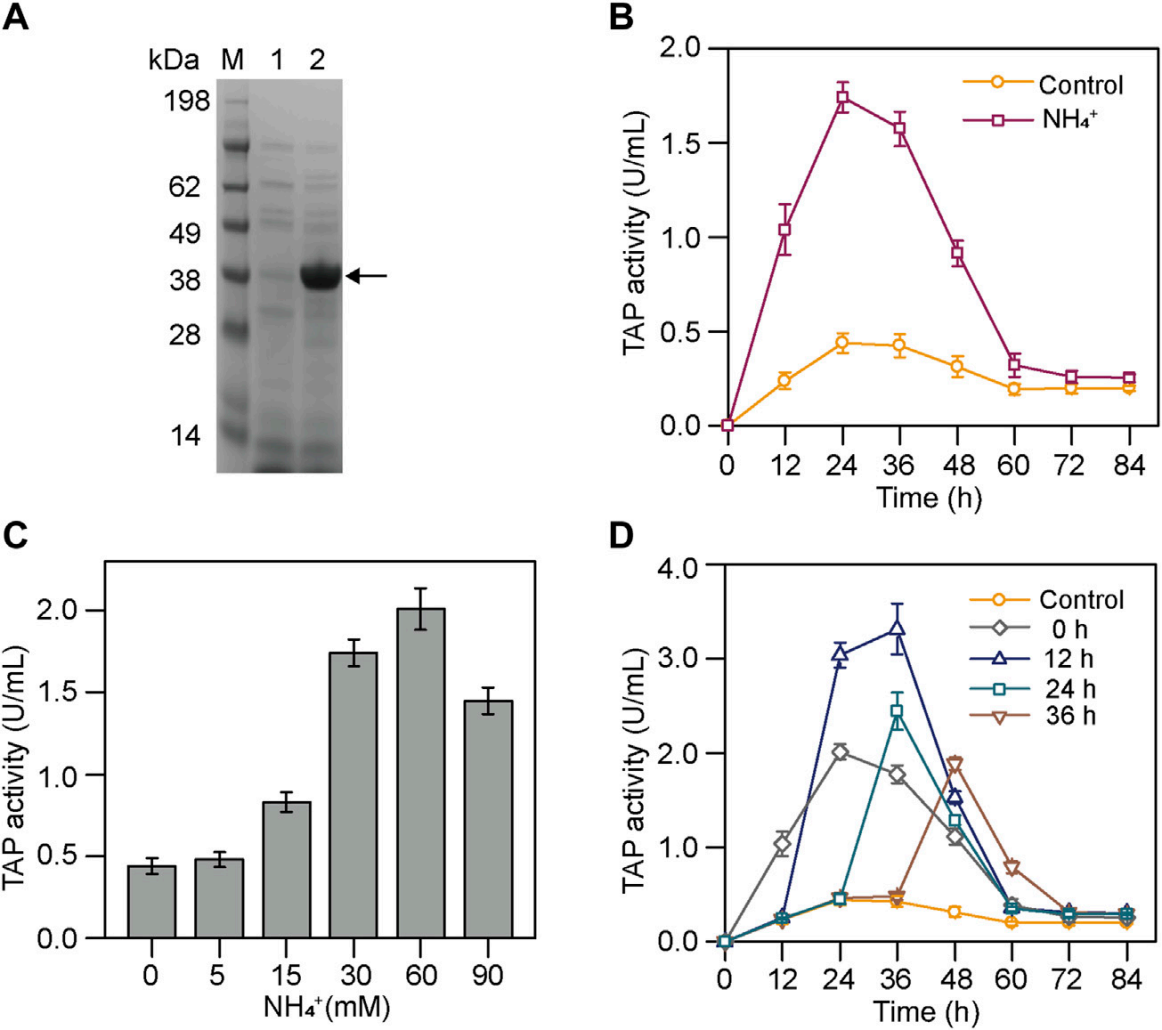
Effects of NH_4_^+^ on the TAP activity in smY2019∆*tg*. **(A)** SDS-PAGE analysis of culture supernatant after cultivation for 84 h. Lane 1: smY2019∆*tg*; lane 2: smY 2019. The TGase band is indicated with single arrows. **(B)** Time course of TAP activity in smY2019∆*tg* with or without 30 mM NH_4_^+^ addition. **(C)** Effect of NH_4_^+^ concentration on the TAP activity in smY2019∆*tg*. The culture supernatant from the cultivation for 24 h was used for TAP activity determination. **(D)** Effect of NH_4_^+^ addition time on TAP activity during the culture process at a constant NH_4_^+^ concentration of 60 mM. NH_4_^+^ was added in the form of (NH_4_)_2_SO_4_.

It has shown that the biosynthesis of pro-TGase is simultaneous with its activation at the first half of *S. mobaraensis* fermentation, and the inactivation of the mature TGase could be seen in the later stage of the fermentation (Figure 1A and Supplementary Figure S1). Therefore, it is hard to characterize TAP activity using *S. mobaraensis* with TGase production. It is essential to establish a method to measure the TAP activity accurately. The pro-TGase from *S. mobaraensis* was expressed in *E. coli* BL21 (DE3) and purified (Supplementary Figure S2A). The TAP activity was measured using the purified pro-TGase as a substrate and indicated by TGase activity. Meanwhile, the previously constructed TGase-deficient strain smY2019∆*tg* was used as the research host for analyzing TAP activity during fermentation (Yin et al., 2021). smY2019∆*tg* did not produce TGase under the same cultivation condition, eliminating the interference of its own TGase activity on TAP activity measurement (Figure 2A). Compared with the previous method, this method exclusively reflects the activity of protease that can activate pro-TGase, which was more sensitive and reliable (Zhang et al., 2012).

We here analyzed the TAP activities of smY2019∆*tg* in the absence and presence of (NH_4_)_2_SO_4_. The TAP activity in smY2019∆*tg* with 30 mM NH_4_^+^ addition grew much faster than that of the cultivation without NH_4_^+^ at the initial 24 h, and the peak value achieved the former reaching 1.74 U/mL, four times higher than that of the latter. Within the next 36 h, the TAP activities under both conditions declined gradually and maintained a very low level (Figure 2B). To further compare the TAP activities, the *in vitro* activation process of samples taken at 24 h was analyzed. The cultivation with NH_4_^+^ completely activated the 0.5 mg/ml pro-TGase within 9 h, while that without NH_4_^+^ did not even activate even after 18 h (Supplementary Figures S2B,C). These results confirmed that NH_4_^+^ increased the TAP activity at the early stage of fermentation. TAMP (purified from surface colonies on plates) was considered to be involved in TGase activation and regulated by SSTI in *S. mobaraensis* (Zotzel et al., 2003a; Juettner et al., 2018; Fuchsbauer, 2021). However, the transcript levels of TAMP and SSTI at 24 h were not changed in the presence and absence of NH_4_^+^ (data not shown). *Streptomyces* are prodigious producers of proteases. (Chater et al., 2010). *Streptomyces coelicolor*, a model organism for the study of *Streptomyces*, contains 56 genes encoding protease, including eight metalloproteinase genes (Bentley et al., 2002). Gene expression often differs when the growth conditions were changed. Adding NH_4_^+^ may induce the expression of a novel metalloproteinase or relieve the inhibition of this activating protease in the early stage of fermentation.

Then, the concentration and time of NH_4_^+^ addition were optimized to further improve the TGase activation. When (NH_4_)_2_SO_4_ was added at the beginning of the fermentation, the TAP activity at 24 h increased with the concentration of NH_4_^+^ from 0 to 60 mM, while further increase in the NH_4_^+^ concentration (90 mM) reduced the protease activity (Figure 2C). The effect of NH_4_^+^ addition time on TAP activity was investigated at a constant NH_4_^+^ concentration (60 mM) during the culture process. After the NH_4_^+^ addition, the TAP activity showed an initial increase followed by a drop in all addition cases (Figure 2D). When NH_4_^+^ was added at 0 or 12 h, the TAP activity increased continuously for 24 h. In contrast, the increase phase was reduced to 12 h in the case of the addition at 24 h or 36 h. Finally, the NH_4_^+^ addition at 12 h achieved the highest TAP activity among the tested addition time. To be noted, SSTI bands at 72 h were smaller than those at 36 h (Figure 1D). Researchers have demonstrated that SSTI is secreted into the fermentation medium in an early cultivation stage and partially degraded by tripeptidyl aminopeptidase in the later stage (Juettner et al., 2020). Generally, partial degradation endows SSTI with full TAP inhibitory activity (Juettner et al., 2020). Thus, SSTI might undergo a similar processing, which may account for the rapid decrease in TAP activity in the later phase of fermentation.

### Enhance the Productivity of TGase by Regulating Zymogen Activation

**FIGURE 3:**
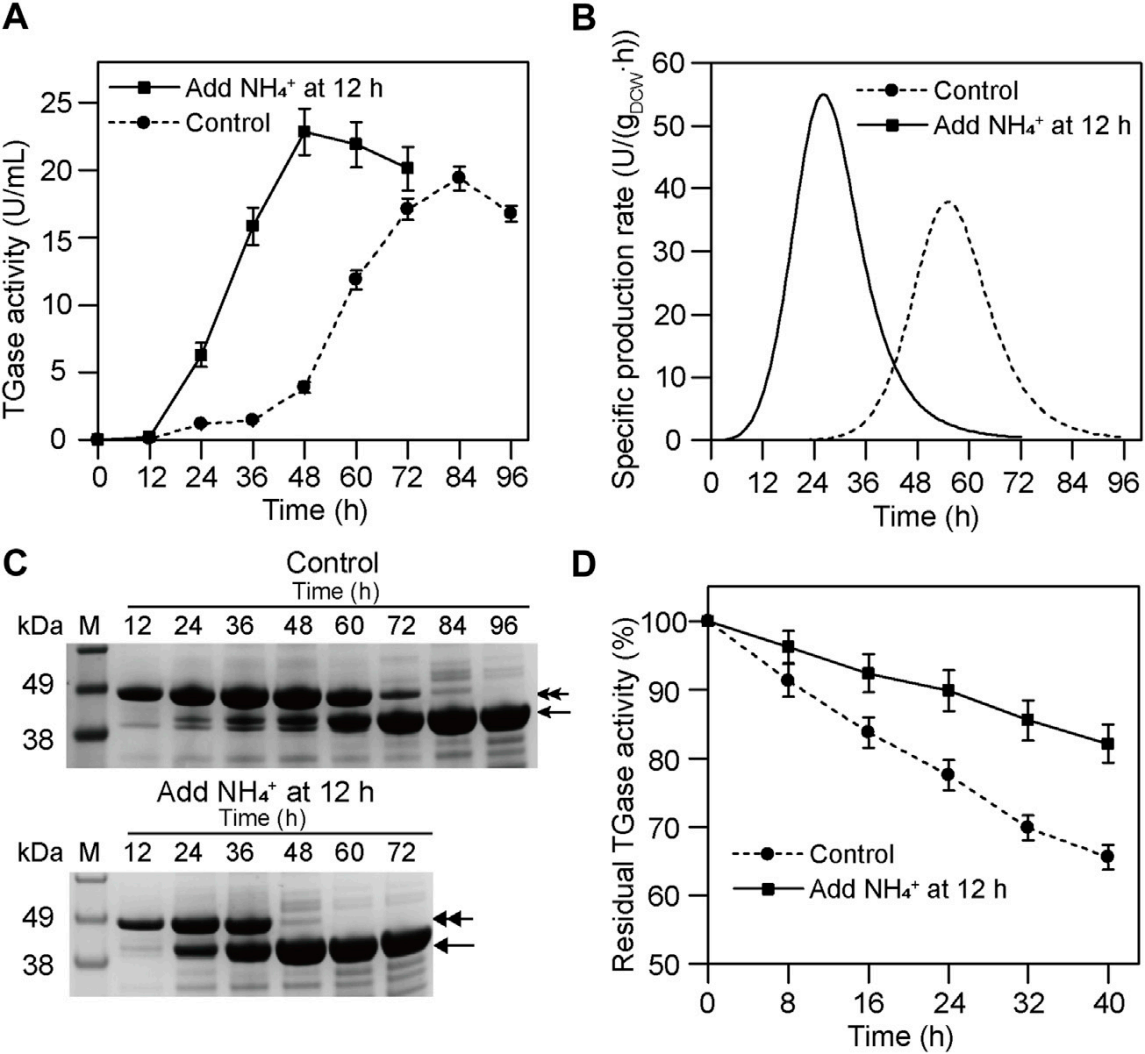
Effects of NH_4_^+^ on TGase production by smY 2019. **(A)** Time course of TGase production. **(B)** Specific production rate curve of TGase. **(C)** SDS-PAGE analysis of the culture supernatant. The pro-TGase and TGase bands are indicated with double and single arrows, respectively. **(D)** Storage stability of the crude TGase solution at room temperature. The crude TGase solution referred to 48-h culture supernatant with 60 mM NH_4_^+^ addition. The 84-h culture supernatant without addition of NH_4_^+^ was used as a control. NH_4_^+^ was added in the form of (NH_4_)_2_SO_4_.

To improve TGase productivity, NH_4_^+^ (60 mM) was added at 12 h during the fermentation of smY 2019. As shown in Figure 3A, the fermentation with NH_4_^+^ addition achieved the peak value of TGase activity at 48 h, 36 h earlier than the fermentation without NH_4_^+^ addition. Accordingly, the peak of the specific production rate also shifted forward when NH_4_^+^ was added (Figure 3B). As indicated by SDS-PAGE analysis, the pro-TGase band was completely converted into the mature TGase band at 48 h in the case of the NH_4_^+^ addition, and this band conversion ended at 84 h without NH_4_^+^ addition (Figure 3C). Moreover, the TGase yield of the former was 18% higher than that of the latter (Figure 3A). Finally, through the NH_4_^+^ addition, TGase productivity was increased from 0.23 U/(mLh) to 0.48 U/(mLh). In a previous study, MgCl_2_ had been shown to have a positive effect on the activation of pro-TGase (Zhang et al., 2012). It is noteworthy that this activation was investigated in the context of a small amount of zymogen. For smY 2019, a high-yielding pro-TGase mutant, the activation effect was not improved after optimizing the amount of MgCl_2_ (Supplementary Figure S3). Excessive addition of MgCl_2_ could even be deleterious for the growth of *S. mobaraensis* (Zhang et al., 2012). As a nitrogen source, NH_4_^+^ was harmless to cell growth and was a more appropriate activator of pro-TGase.

To be noted, NH_4_^+^ addition improved the storage stability of the crude TGase solution of smY 2019. When treated at room temperature for 40 h, the TGase activity of the culture supernatant from the 48-h culture broth with NH_4_^+^ addition retained 82% of initial activity, while that from 84-h culture broth without NH_4_^+^ only obtained 65% residual activity (Figure 3D). This is probably due to the fact that a lot of proteases were produced at the later stage of *S. mobaraensis*, resulting in proteolytic degradation of TGase (Fuchsbauer, 2021). Therefore, the reduced fermentation period could not only increase the economy of the TGase but also its storage stability.

## Conclusions

This study was the first to demonstrate that NH_4_^+^ addition was capable to enhance TAP activity in *S. mobaraensis*. After optimizing the amount and time of NH_4_^+^ added, pro-TGase activation was considerably improved as TAP activity was significantly enhanced. Hence, the TGase productivity was increased 2.1 times relative to that without NH_4_^+^ addition, and maximum production was obtained in 43% less time. Our study makes the production of TGase more cost-effective and enhances the storage stability of crude enzyme solutions. Future work is to explore the key protease by transcriptomic comparison and regulate its expression at the gene level to efficiently activate pro-TGase using an inexpensive medium.

## Data Availability

The original contributions presented in the study are included in the article/Supplementary Material, further inquiries can be directed to the corresponding author.
