# Supplementary material for: Improved Productivity of Streptomyces mobaraensis Transglutaminase by Regulating Zymogen Activation
Source: Front Bioeng Biotechnol. 2022 Apr 14;10:878795. doi: 10.3389/fbioe.2022.878795 (PMC9047793; doi:10.3389/fbioe.2022.878795)
Supplement: Supplementary file 1 [file DataSheet1.docx]

**Supplementary materials**


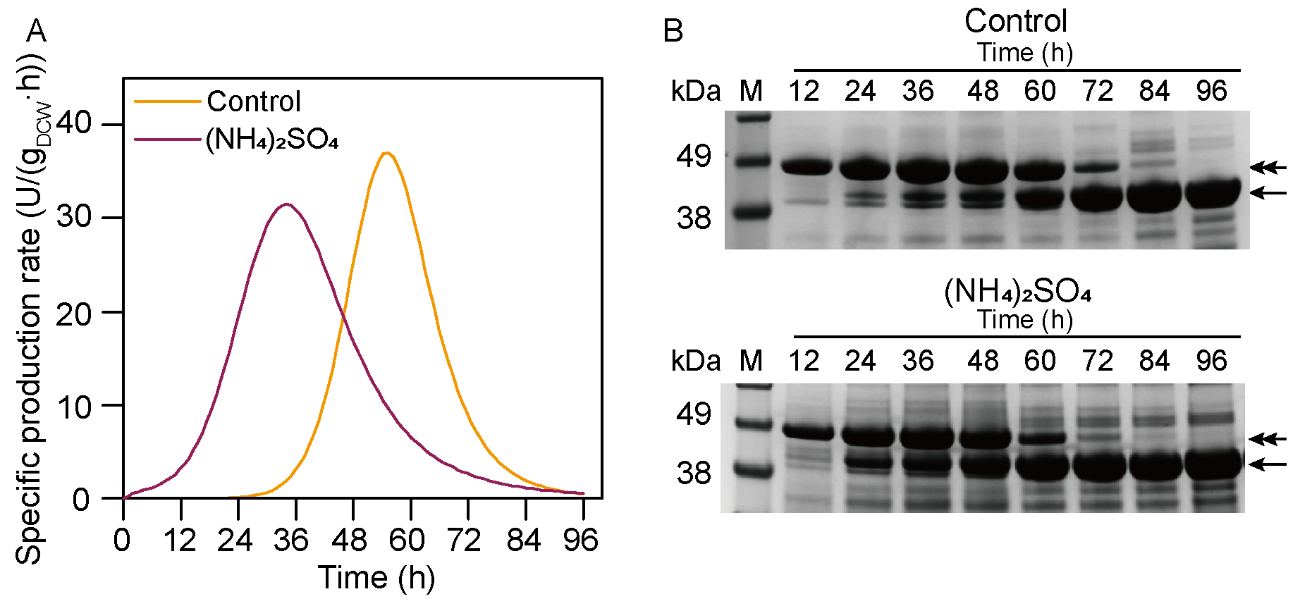


Figure S1 Effects of (NH_4_)_2_SO_4_ on TGase activation. (A) The specific production rate curve of TGase. (B) SDS-PAGE analysis of the culture supernatants. The pro-TGase and TGase bands are indicated with double and single arrows, respectively. Control: without adding NH_4_^+^, (NH_4_)_2_SO_4_: adding 30 mM NH_4_^+^. NH_4_^+^ was added in form of (NH_4_)_2_SO_4_.


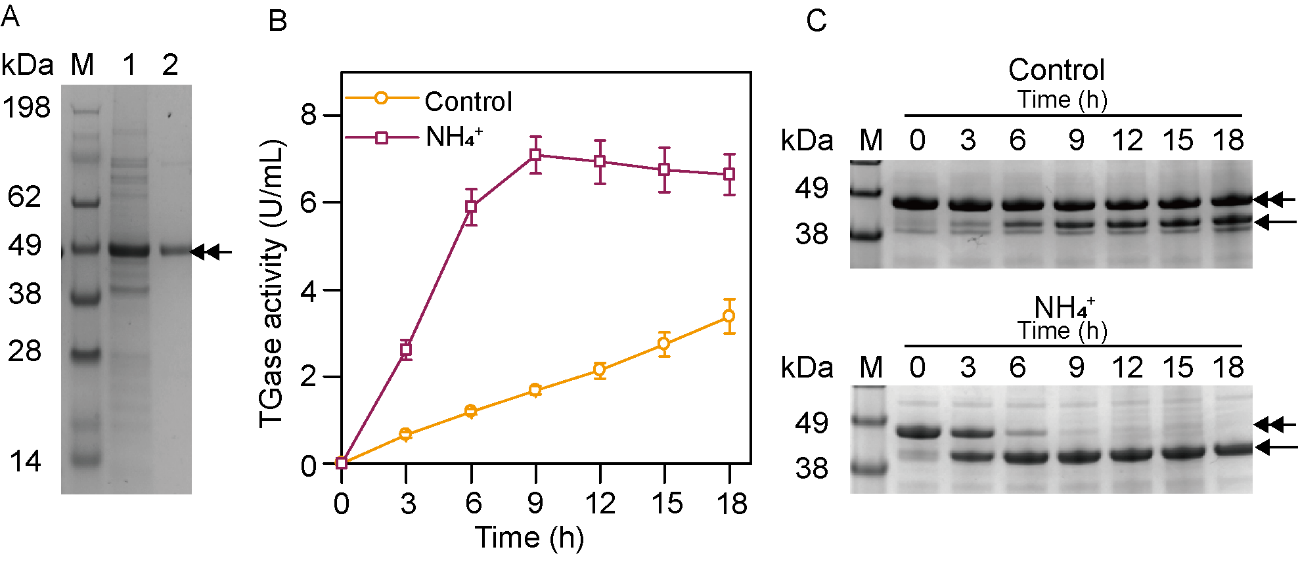


Figure S2 Activation process analysis of the pro-TGase *in vitro*. (A) Expression and purification of pro-TGase in *E. coli* BL21(DE3). Lane M, protein marker; lane 1, the culture supernatant after cultivation for 30 h; lane 2, the purified pro-TGase. The pro-TGase band is indicated with double arrow. (B) Changes in TGase activity in mixed reaction solution. (C) SDS-PAGE analysis of pro-TGase activation process. The reaction was conducted by mixing equal volumes of purified pro-TGase solution (0.5 mg/mL) and 24 h-fermentation supernatant of smY2019∆*tg* at 30℃. The pro-TGase and TGase bands are indicated with double and single arrows, respectively. Control: without adding NH_4_^+^, NH_4_^+^: adding 60 mM NH_4_^+^.


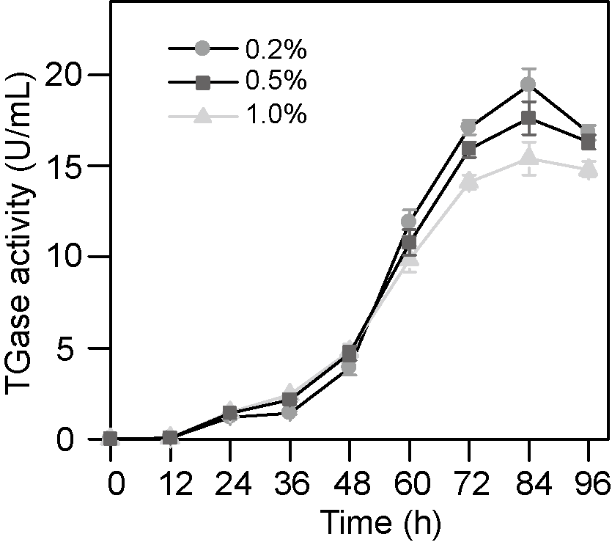


Figure S3 Effects of the amount of MgCl_2_ on TGase production by smY2019.
